# Aged Garlic Extract (AGE) and Its Constituent S-Allyl-Cysteine (SAC) Inhibit the Expression of Pro-Inflammatory Genes Induced in Bronchial Epithelial IB3-1 Cells by Exposure to the SARS-CoV-2 Spike Protein and the BNT162b2 Vaccine

**DOI:** 10.3390/molecules29245938

**Published:** 2024-12-16

**Authors:** Jessica Gasparello, Chiara Papi, Giovanni Marzaro, Alberto Macone, Matteo Zurlo, Alessia Finotti, Enzo Agostinelli, Roberto Gambari

**Affiliations:** 1 Department of Life Sciences and Biotechnology, Ferrara University, 44121 Ferrara, Italy; jessica.gasparello@gmail.com (J.G.); chiara.papi@unife.it (C.P.); matteo.zurlo@unife.it (M.Z.); alessia.finotti@unife.it (A.F.); 2 Department of Diagnostics and Public Health, University of Verona, 37134 Verona, Italy; giovanni.marzaro@univr.it; 3 Department of Biochemical Sciences ‘A. Rossi Fanelli’, Sapienza University of Rome, 00185 Rome, Italy; alberto.macone@uniroma1.it; 4 Department of Sensory Organs, Sapienza University of Rome, Policlinico Umberto I, Viale del Policlinico 155, 00161 Rome, Italy; 5 International Polyamines Foundation ‘ETS-ONLUS’, Via del Forte Tiburtino 98, 00159 Rome, Italy

**Keywords:** garlic, natural products, Toll-like receptor 4, NF-κB, pro-inflammatory genes

## Abstract

Garlic (*Allium sativum* L.) is a species of the onion family (*Alliaceae*) widely used as a food and a folk medicine. The objective of this study was to determine the effects of AGE (aged garlic extract) on pro-inflammatory genes relevant to COVID-19. To this aim, we treated bronchial epithelial IB3-1 cells with SARS-CoV-2 spike protein (S-protein) or with the COVID-19 BNT162b2 vaccine in the absence or in the presence of AGE. The results obtained demonstrated that AGE is a potent inhibitor of the S-protein-induced expression of the IL-1β, IL-6 and IL-8 genes. Bio-Plex analysis demonstrated that AGE reduced release of IL-6 and IL-8, which were highly induced by S-protein. No inhibition of cells’ growth, toxicity and pro-apoptotic effects were found in AGE-treated cells. The effects of one of the major AGE constituents (S-allyl cysteine, SAC) were studied on the same experimental model systems. SAC was able to inhibit the S-protein-induced expression of IL-1β, IL-6 and IL-8 genes and extracellular release of IL-6 and IL-8, confirming that S-allyl-cysteine is one of the constituents of AGE that is responsible for inhibiting S-protein-induced pro-inflammatory genes. Docking experiments suggest that a possible mechanism of action of SAC is an interference with the activity of Toll-like receptors (TLRs), particularly TLR4, thereby inhibiting NF-κB- and NF-κB-regulated genes, such as IL-1β, IL-6 and IL-8 genes. These results suggest that both AGE and SAC deserve further experimental efforts to verify their effects on pro-inflammatory genes in SARS-CoV-2-infected cells.

## 1. Introduction

Garlic (*Allium sativum* L.) is a species of the onion family (*Alliaceae*), which is not only used as a food supplement but also as a medicinal plant in folk medicine [1,2,3,4,5]. A number of epidemiological studies have strongly suggested that garlic-based products are effective in the prevention and treatment of several human diseases. In fact, garlic-based products exhibit multiple pharmacological properties, such as anticarcinogenic, antithrombotic, hypolipidemic and hepatoprotective activities [6,7,8,9]. For instance, an interesting finding is that this herb exerts cytotoxic effects on multidrug-resistant human cancer cells by altering mitochondrial permeability [10] and inducing apoptosis [11]. 

Among a large variety of commercial garlic-based products, AGE (aged garlic extract) is well known and has been studied in detail [10]. AGE is a commercially available odorless preparation obtained by immersing fresh garlic in 15% aqueous ethanol solution over a prolonged period of time (up to 20 months) at room temperature [10,12,13,14,15]. This natural product has been shown to possess immunomodulatory and anticancer properties [10]. Several of the beneficial effects of garlic have been attributed to several bioactive compounds, including the lipid-soluble allyl sulfur compounds (e.g., diallyl sulfide, diallyl disulfide and diallyl trisulfide) and water-soluble compounds, such as S-allyl-cysteine (SAC), S-allylmercaptocysteine (SAMC) and S-1-propenylcysteine (S1PC) [16,17,18]. These bioactive compounds might be extracted from AGE by unique manufacturing processes [11]. 

Most of the available data support the concept that AGE and AGE-related compounds retain anti-inflammatory properties. This is highly relevant for a possible treatment of the pandemic coronavirus disease 2019 (COVID-19) since inflammatory complications are a key factor determining the severity of COVID-19 [19,20,21,22]. In fact, it is firmly established that COVID-19, caused by severe acute respiratory syndrome coronavirus 2 (SARS-CoV-2), is characterized by two major clinical phases: (a) a SARS-CoV-2 infection of target cells and tissues and (b) a deep inflammatory state, known as a “cytokine storm” [21,22,23,24]. In this respect, the SARS-CoV-2 spike (S-protein) plays a very important role. The S-protein, through interactions with cellular receptors, is responsible for deep cellular changes, including the hyperactivation of nuclear factor kappa-B (NF-κB) by the IL-6/STATs axis [25]. This induces the acute respiratory distress syndrome (ARDS), frequently observed in severe COVID-19 patients [26], and is clearly associated with the severity of the pathology [26,27,28]. 

The impact of anti-inflammatory protocols for anti-SARS-CoV-2 pharmacological strategies is clear, as recently demonstrated by the effective treatments targeting interleukin-6 (IL-6) [29] and interleukin-8 (IL-8) [30]. Notably, the pharmacological approach for treating ARDS steadily needs novel anti-inflammatory reagents as different COVID-19 patients might respond in a different way to these treatments [31]. Several anti-inflammatory strategies to reduce the COVID-19 “cytokine storm” and associated ARDS have been proposed using biomolecules derived from herbal medicinal extracts and have been reviewed by several authors [32,33]. This was judged to be a key strategy at the beginning of the pandemic in consideration of the unknown nature of the disease and the lack of effective treatment protocols and approved vaccines [34,35]. Repurposing of known plant-derived reagents for anti-inflammatory activity against a COVID-19 “cytokine storm” might be of great interest [36,37]. 

The main objectives of the present study were (a) to verify whether AGE and SAC inhibit the expression of pro-inflammatory genes relevant in COVID-19 and (b) to identify a possible mechanism(s) of action of SAC. To this aim, human bronchial epithelial IB3-1 cells [38] have been exposed to the SARS-CoV-2 spike protein [38,39] or to the spike mRNA-based BNT162b2 vaccine [40,41] in order to induce hyperexpression of pro-inflammatory genes; this experimental model system has been proved useful to screen and characterize anti-inflammatory agents, such as sulforaphane [38] and trimethyl-angelicin [42]. 

In order to characterize the effects of AGE and SAC, IB3-1 cells treated with the S-protein or the BNT162b2 vaccine were then cultured in the presence of AGE and SAC. After 72 h, the cells were harvested and RNA isolated for RT-qPCR analysis focusing on the expression of the pro-inflammatory genes IL-1β, IL-6 and IL8. In addition, supernatants were taken for analysis of the secreted proteins using a Bio-Plex-based analysis [38,39].

## 2. Results

### 2.1. The Experimental Model System: Induction of Pro-Inflammatory Genes in Bronchial Epithelial IB3-1 Cells After Exposure to the SARS-CoV-2 Spike Protein and the COVID-19 BNT162b2 Vaccine

**Figure 1:**
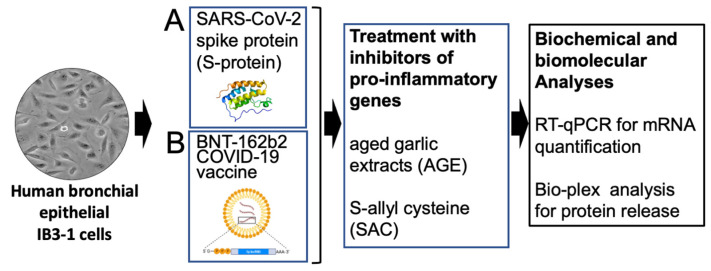
Pictorial representation of the experimental model systems of the present study. A: human bronchial epithelial IB3-1 cells stimulated with the SARS-CoV-1 spike protein; B: IB3-1 cells stimulated with the BNT162b2 vaccine.

Two experimental model systems have been considered in the present study, with both of them exhibiting the ability to express at high levels several pro-inflammatory genes. The first is constituted by human bronchial epithelial IB3-1 cells exposed to the SARS-CoV-2 spike protein and elsewhere described in detail [38,39]. The exposure to S-protein leads to a NF-κB-dependent increase in the expression of pro-inflammatory genes, such as those coding interleukin-6 (IL-6), IL-8 and IL-1β. This experimental system (Figure 1A) has been already employed for studying the effects of sulforaphane (SFN) [38] and agomiR-93-5p [39] on the expression of pro-inflammatory genes [38,39]. The second experimental model system (Figure 1B) is based on the treatment of IB3-1 cells with the COVID-19 BNT162b2 vaccine [40,41]. In our present study, IB3-1 cells have been treated with 5 nM S-protein or 1 μg/mL of BNT162b2 unless otherwise stated. These are the lowest concentrations of S-protein and BNT162b2 necessary to obtain efficient and highly reproducible induction of IL-1β, IL-6 and IL-8 proteins by treated IB3.1 cells, according to Gasparello et al. [38]. These treatments were performed in the absence or in the presence of increasing concentrations of AGE and SAC. After treatments for 72 h, RNA was purified from the treated cells for RT-qPCR analysis, and cellular supernatants were isolated for the analysis of secreted cytokines, chemokines and growth factors in order to identify alteration(s) of the SARS-CoV-2 spike protein-induced secretome profile.

The spike-induced experimental model system (approach “A” of Figure 1) has been presented in two studies by Gasparello et al. [38,39]. In both studies the recombinant spike 139 KDa protein fragment was demonstrated to be able to induce the expression of pro-inflammatory genes, such as those coding IL-6, IL-8, IL-1β, G-CSF and GM-CSF. This was studied by analyzing the relative mRNA content by RT-qPCR and the protein release by Bio-Plex technology. This effect of the S-protein has been reported by other groups using experimental model systems closely related to the cells of the immune system (T cells, macrophages, monocytes, dendritic cells and endothelial cells) [43,44,45] primarily involved in the production of the inflammatory cytokines and chemokines of the COVID-19 “cytokine storm”. For instance, Khan et al. observed that the spike (S) protein potently induced inflammatory cytokines and chemokines, including IL-6, IL-1β and IL-8, in human and mouse macrophages [46]. Interestingly, and in agreement with the results of our study, human lung epithelial cells also produced inflammatory cytokines and chemokines when stimulated with extracellular S-protein [46].

In the present study, we employed the complementary approach “B” (Figure 1), based on the exposure of IB3-1 cells to the spike (mRNA)-based BNT162b2 vaccine. Treatment of IB3-1 cells with the BNT162b2 vaccine was associated with high production and release of full-length spike 180 kDa protein, as demonstrated by Western blotting and ELISA assays. Interestingly, the BNT162b2-based experimental model system proved to be very useful for our studies, as it showed a higher induced expression of pro-inflammatory proteins compared to the same IB3-1 cells treated with the recombinant spike protein. This is shown in Supplementary Figure S1, reporting a comparison between the two approaches, based on the analysis of the expression of the IL-8 gene. We would like to underline that our experimental model systems lend themselves very well to the screening of potential molecules capable of inhibiting the expression of pro-inflammatory genes rather than to the study of the cellular and molecular bases of the activation of a COVID-19 “cytokine storm”.

### 2.2. GC-MS Analysis of AGE and SAC

**Figure 2:**
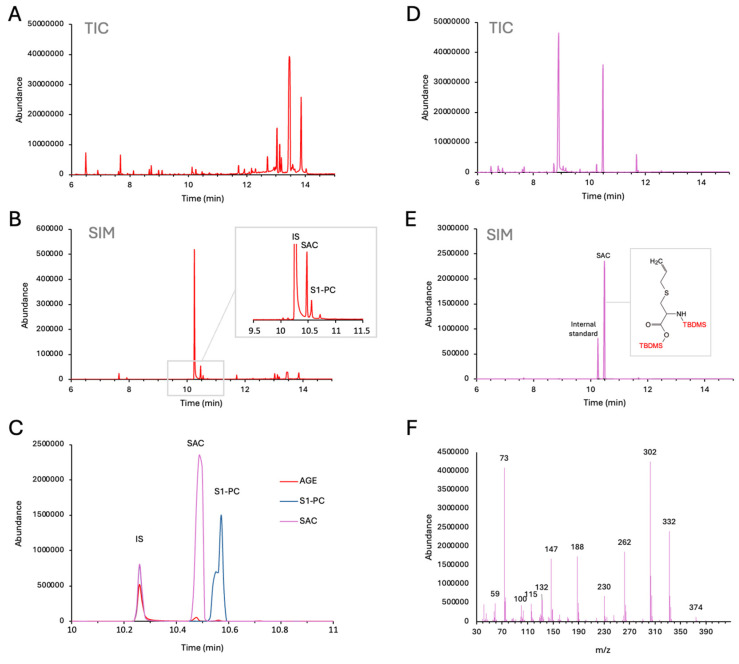
GC-MS analysis of AGE and SAC. (**A**) Total ion current (TIC) chromatogram of the AGE sample derivatized with TBDMS; (**B**) total ion current (TIC) chromatogram of the SAC sample derivatized with TBDMS; (**C**) overlaid selected ion chromatograms (SIM) of AGE and SAC samples derivatized with TBDMS acquiring the following ions: *m*/*z* 239 (3,4-dimethoxybenzoic acid, internal standard) and *m*/*z* 332 (SAC); (**D**), electron impact mass spectrum of S-allyl-cysteine as di-TBDMS derivative (retention time 10.49 min). Both AGE (**A**–**C**) and the SAC (**D**–**F**) powders were freshly dissolved in complete RPMI-1640 medium prior to each experiment. Enlargements/details are shown in the sub-panels in (**B**,**E**).

For chemical characterization, AGE and SAC were analyzed by GC-MS as TBDMS derivatives described in detail in the Supplementary Materials. Figure 2 displays the GC-MS analysis of AGE (Figure 2A–C) and SAC (Figure 2D–F). In both figures, total ion chromatogram (TIC) and selected ion monitoring (SIM) modes are shown. By performing this GC-MS analytical study, we were interested in qualitative analysis of the aged garlic extract employed in our biological experiments, focusing on identifying its chemical constituents, including S-allyl-cysteine (SAC).

These analyses reveal the presence of SAC within the aged garlic extracts powder, as evidenced by the overlaid SIM chromatograms (Figure 2C). SAC, as a di-TBDMS derivative, elutes at 10.49 min (Figure 2C, inset), and its identity is confirmed by the EI-mass spectrum (Figure 2D), wherein the loss of the t-butyl group yields a characteristic [M–t-Bu]+ ion (*m*/*z* 332). As expected, the presence of S-1-propenyl-l-cysteine (S1PC) was also noted in the AGE solution (Figure 2C).

### 2.3. The Treatment of IB3-1 Cells with AGE Reverses Upregulation of Pro-Inflammatory Genes Induced by the SARS-CoV-2 Spike Protein

**Figure 3:**
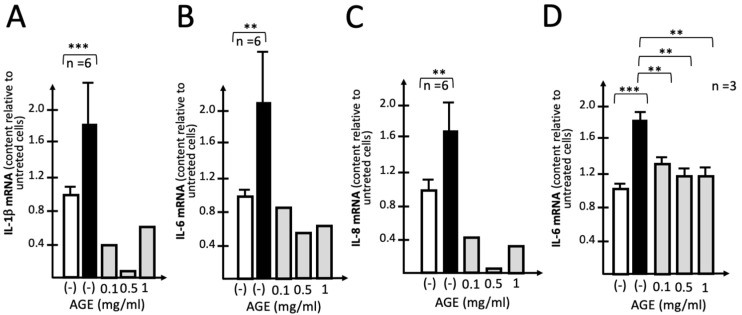
Representative examples of the effects of increasing amounts of AGE on S-protein-induced expression of IL-1β (**A**), IL-6 (**B**) and IL-8 (**C**) genes. IB3-1 cells were either untreated (white histograms) or exposed to SASRS-CoV-2 S-protein (5 nM) in the absence (black histograms) or in the presence of the indicated concentrations of AGE (grey histograms). After 72 h, total RNA was isolated and mRNA content analyzed by RT-qPCR. (**D**) Summary of experiments (*n* = 3) analyzing the relative content of IL-6 mRNA. Results represent the average ± S.D. Statistical significance was high: ** (*p* < 0.01) and *** (*p* < 0.001).

In the experiment shown in Figure 3, the effects of increasing amounts of AGE were studied on the S-protein-induced expression of IL-1β, IL-6 and IL-8 genes. This experiment is the proof of principle demonstrating that treatment with 0.1–0.5 mg/mL of AGE leads to a sharp inhibition of the expression of IL-1β (Figure 3A), IL-6 (Figure 3B) and IL-8 (Figure 3C) genes. This finding was highly reproducible and a summary of independent replicates analyzing the relative content of IL-6 mRNA is shown in Figure 3D. Analysis of IL-6 gene expression was selected because this gene is, at least in our hands, the most upregulated after S-protein stimulation of IB3-1 cells (Supplementary Figure S2 and Table S1). The use of higher concentrations of AGE were avoided since it is known that this extract is able to induce also pro-apoptotic effects at high concentrations due to a mitochondrial membrane depolarization process already observed in other cell types [10,11]. It should be noted that 0.5 mg/mL AGE fully suppressed IL-6 upregulation due to stimulation with 5 nM S-protein. No major effects of AGE on IB3-1 cell growth were observed.

### 2.4. Effects of AGE on Toxicity and Apoptosis

**Figure 4:**
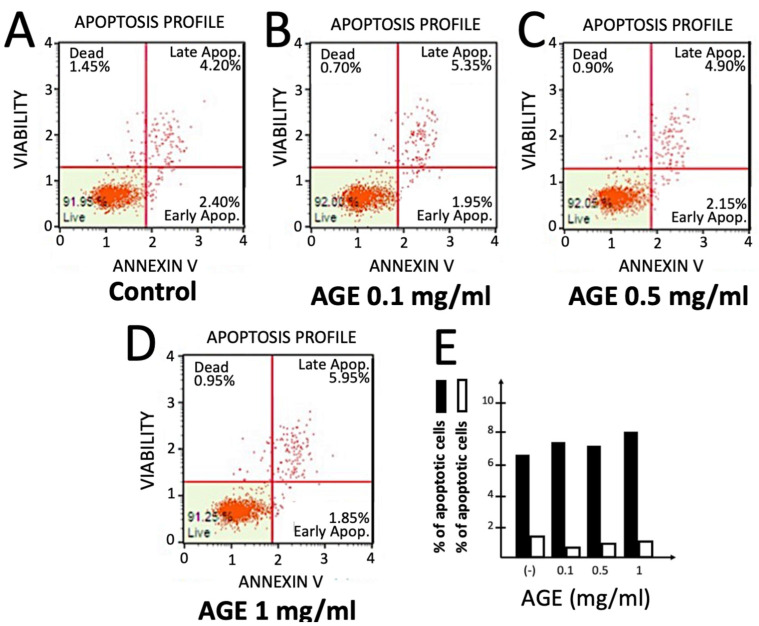
Effects of AGE on cell vitality and apoptosis. IB3-1 cells were cultured in the absence or the presence of the indicated concentrations of AGE for 72 h. Vitality and apoptosis were determined using the Muse Annexin V & Dead Cell Kit. (**A**–**D**). Representative annex V assays performed on control untreated IB3-1 cells (**A**) or IB3-1 cells treated for 72 h with AGE, used at 0.1 mg/mL (**B**), 0.5 mg/mL (**C**) and 1 mg/mL (**D**). A summary of the annexin V assay data is shown in panel (**E**).

The experiment shown in Figure 4 was performed in order to verify whether AGE treatment of IB3-1 cells was to some extent cytotoxic. To this aim, the annexin V assay was employed. This assay differentiates cells in “live cells”, “early apoptotic cells”, “late apoptotic cells” and “dead cells” [47,48]. The results obtained are shown in Figure 4 and demonstrated that AGE, when maintained in contact with IB3-1 cells for 72 h, did not reduce the extent of viable cells and did not induce their apoptosis (see also the summary shown in panel E). In these experiments, IB3-1 cells were cultured for 72 h in the presence of 0.1, 0.5 and 1 mg/mL of AGE.

These data sustain the concept that the inhibitory effects of AGE on the expression of pro-inflammatory genes should not be ascribed to unspecific cytotoxic and/or pro-apoptotic effects, at least at the concentrations used in our experiments. We, however, expect that higher concentrations of AGE might cause some pro-apoptotic effects, considering that AGE and its constituents SAC and S1PC have been shown to exert potential anti-tumor effects caused by activation of the apoptotic pathway [11]. 

### 2.5. The Treatment of IB3-1 Cells with S-Allyl-Cysteine (SAC) Inhibits the Expression of Pro-Inflammatory Genes Induced by SARS-CoV-2 Spike Protein

**Figure 5:**
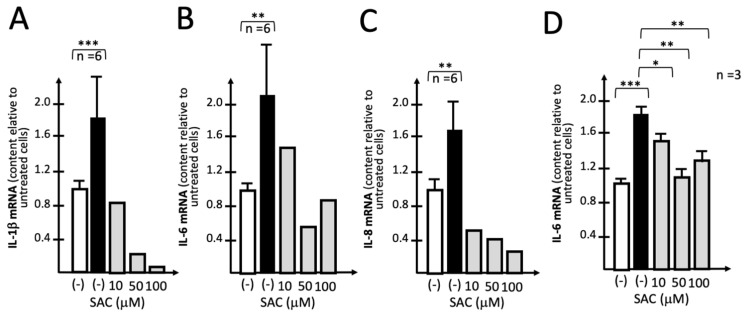
Representative examples of the effects of increasing amounts of SAC on S-protein-induced expression of IL-1β (**A**), IL-6 (**B**) and IL-8 (**C**) genes. IB3-1 cells were either untreated (white histograms) or exposed to SARS-CoV-2 S-protein (5 nM) in the absence (black histograms) or in the presence of the indicated concentrations of SAC (grey histograms). After 72 h, total RNA was isolated and mRNA content analyzed by RT-qPCR. (**D**) Summary of experiments (*n* = 3) analyzing the relative content of IL-6 mRNA. The results represent the average ± S.D. Statistical significance was as follows: * (*p* < 0.05): significant; ** (*p* < 0.01) and *** (*p* < 0.001): highly significant.

During the process of aging of garlic, γ-glutamyl-S-allyl-cysteine is converted to S-allyl-cysteine (SAC) by a γ-glutamyltransferase, as reviewed by Colín-González et al., 2012) [49]. In the experiment shown in Figure 5, the effects of increasing amounts of SAC were studied on the S-protein-induced expression of IL-1β, IL-6 and IL-8 genes. The results obtained indicate that treatment with 10–100 μM SAC leads to a sharp inhibition of the expression of IL-1β (Figure 5A), IL-6 (Figure 5B) and IL-8 (Figure 5C) genes. A summary of experiments analyzing the relative content of IL-6 mRNA, the most upregulated in S-protein-treated IB3-1 cells (Supplementary Figure S1) is shown in Figure 5D.

The data presented in Figure 5 indicate that 10 μM SAC is sufficient to inhibit S-protein-induced expression of the pro-inflammatory genes IL-1β, IL-6 and IL-3, suggesting that S-allyl-cysteine should be considered a compound responsible for the inhibitory effects of AGE on pro-inflammatory genes. An inhibitory effect higher that 50% was usually achieved with 50 μM SAC, which was considered an optimal concentration to be used in subsequent experiments.

### 2.6. Effects of AGE and SAC on the Release of IL-6 and IL-8 by IB3-1 Cells Induced to Express Pro-Inflammatory Genes by Exposure to SARS-CoV-2 S-Protein

We have previously reported that S-protein induces IB3-1 cells to release higher levels of cytokines, chemokines and growth factors when compared to control untreated cells. This can be studied by Bio-Plex technology that allows the parallel analysis of 27 different released proteins, including cytokines, chemokines and growth factors [50]. We have also previously published that IL-1β is expressed at very low levels in IB3-1 cells [38,39]. For this reason, we limited the analysis of secreted proteins to IL-6 and IL-8. 

**Figure 6:**
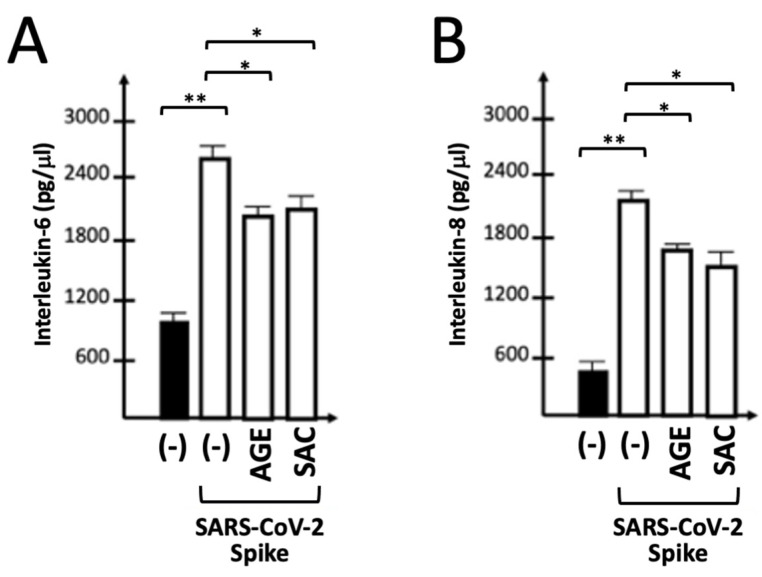
Effects of AGE and SAC on release of IL-6 (**A**) and IL-8 (**B**) by S-protein-stimulated IB3-1 cells. A panel of 27 cytokines/chemokines/growth factors was analyzed by Bio-Plex technology in IB3-1-infected cells 24 h after exposure to the S-protein. Results represent the average ± SD (*n* = 3). The results represent the average ± S.D. Statistical significance was as follows: * (*p* < 0.05): significant; ** (*p* < 0.01): highly significant.

Figure 6 shows that treatment with both AGE (0.5 mg/L) and SAC (50 μM) leads to inhibition of the release of IL-6 (Figure 6A) and IL-8 (Figure 6B) by IB3-1 stimulated with 5 nM S-protein. These concentrations of AGE and SAC were shown to induce a high level of inhibition (usually more than 50%) of spike-induced expression of IL-1β, IL-6 and IL-8, as outlined in Figure 3 and Figure 5. The Bio-Plex assay was performed on supernatants isolated from 72 h treated IB3-1 cell cultures, either untreated or treated with the S-protein in the presence of AGE and SAC. 

### 2.7. Effects of AGE and SAC on the Expression of Pro-Inflammatory Genes Induced by the BNT162b2 COVID-19 Vaccine

In the experiments based on the use of the BNT162b2 vaccine, the effects of AGE and SAC were studied on the expression of the IL-8 gene. This was performed because IL-8 gene expression was induced by the BNT162b2 vaccine at very high levels compared to the S-protein induction (Supplementary Figure S2). As expected, the intracellular content of SARS-CoV-2 spike protein mRNA increases depending on the employed concentrations in BNT-162b2-treated IB3-1 cells. A significant increase (*p* < 0.01) was observed when the 1 μg/mL vaccine was used. 

As expected from previously published observations [40,41], production of S-protein was detectable when Western blotting was performed using cellular lysates from IB3-1 cells treated with the BNT162b2 vaccine. As expected from the notion that in many cellular systems the S-protein induces the expression of pro-inflammatory genes through upregulation of NF-κB [51,52,53], an increase in the expression of NF-κB was found in IB3-1 cells treated with the BNT-162b2 vaccine. 

**Figure 7:**
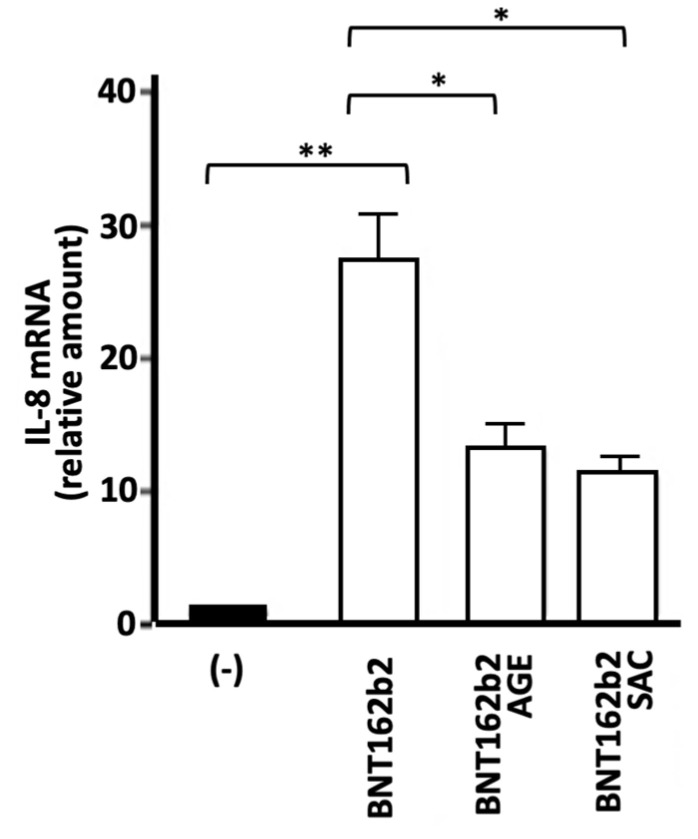
Effects of AGE and SAC on IL-8 gene expression induced in IB3-1 cells by treatment with BNT162b2 COVID-19 vaccine. The results represent the average ± S.D (*n* = 3). Statistical significance was as follows: * (*p* < 0.05): significant; ** (*p* < 0.01): highly significant.

In agreement with the NF-κB upregulation, Figure 7 shows that treatment of IB3-1 cells with the BNT162b2 vaccine causes >30-fold increase in the content of IL-8 RNA. This increase was much lower (1.5–3-fold with respect to control untreated IB3-1 cells) when the SARS-CoV-2 S-protein was employed to induce an NF-κB-dependent increase in the expression of pro-inflammatory genes [38,39]. In agreement with the findings reported in Figure 3 and Figure 5, treatment of IB3-1 cells with AGE or SAC inhibits the expression of the IL-8 gene induced by the BNT162b2 vaccine. 

### 2.8. S-Allyl-Cysteine (SAC) Efficiently Interacts with Toll-like Receptor 4 (TLR4): Molecular Docking and Molecular Dynamics Analyses

Preliminary analyses demonstrated a lack of binding of SAC to NF-κB. Binding of low-molecular-weight drugs were reported by our group studying trimethylangelicin and analogues [42], corilagin [54] and sulforaphane [51]. In these cases, efficient interactions with NF-κB were found. On the contrary, no evidence of molecular interaction between SAC and NF-κB was found in our docking analyses.

Since Toll-like receptors are upstream regulators of the NF-κB signaling [55,56,57,58], the interaction between SAC and Toll-like receptors was considered. The computational studies conducted in this work considered only TLR4 as potential target for SAC since for this member of TLRs a reliable 3D structure was available. Unfortunately, for other TLR heterodimers (e.g., TLR2/TLR1, TLR2/TLR6), the complex geometries have not yet been clarified, and it is still not clear whether in these heterodimers the TIR domains interact through a “face-to-face” geometry (as in the case of a TLR4 homodimer) or a “back-to-face” geometry. Deeper investigation into the potential interaction of SAC with other TLR members is currently ongoing in our research groups. The docking simulations employ the well-known docking software AutoDock Vina (v 1.2.0) [59]. TLR4 is clearly involved in spike-mediated activation of NF-κB and inflammation [60,61,62,63]. The interplay of TLR4 and SARS-CoV-2, contributing to the complex mechanisms of inflammation and severity in COVID-19 infections, has been recently reviewed by Asaba et al. [63]. Interestingly, an aptamer blocking SARS-CoV-2 spike–TLR4 interaction selectively inhibits SARS-CoV-2-induced inflammation [64], strongly suggesting that, in addition to TRL2, TLR4 should be considered a biochemical target for COVID-19 therapy [64,65]. We were also interested in studying TLR4 considering the recent report suggesting that TLR4 recognizes the BNT162b2 vaccine’s empty lipid nanoparticle to induce NF-κB [66]. 

**Figure 8:**
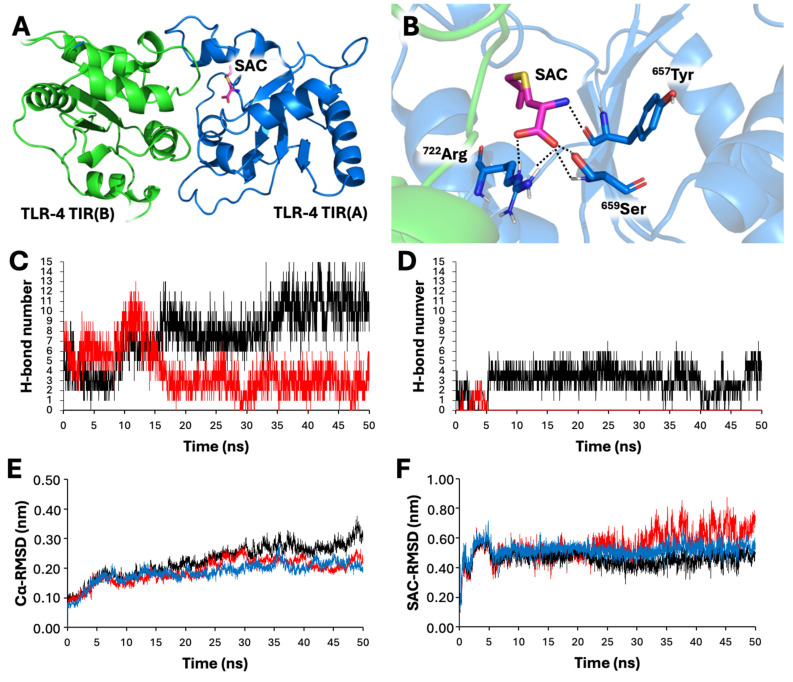
Simulation of SAC binding to TLR-4 through docking and molecular dynamics simulations. The docking studies were conducted with AutoDock Vina [59]. Molecular dynamics studies were conducted with Gromacs software (v. 2024,0). See the Materials and Methods section for further details. Binding mode predicted for SAC (**A**) with the TLR4-TIR dimer and details of interactions (**B**). TLR4 monomers are depicted in blue (chain A) and green (chain B), respectively; SAC is depicted as stick (magenta-colored carbon); hydrogen bonds are depicted as dashed black lines. Number of H bonds formed between chains A and B during 50 ns of MDs (**C**). Black line represents the simulations of apo-TLR-4; red line represents the simulation of SAC-bound TLR-4. Note a marked reduction in interchain interactions as a consequence of the interaction with SAC. Number of H bonds formed between SAC and TLR-4 chain A (black) and chain B (red) (**D**). Root-mean-squared deviation (RMSD, nm) of Cα atoms in SAC-bound TLR-4 (**E**). Black line represents the total RMSD; blue line represents the chain A RMSD; red line represents the chain B RMSD. Root-mean-squared deviation (RMSD, nm) of SAC heavy atoms with respect to Cα atoms (**F**). Black line represents the SAC RMSD relative to the dimer; blue line represents the SAC RMSD relative to chain A; red line represents the SAC RMSD relative to chain B. Note that the binding between SAC and chain A remains constant during the 50 ns of MDs.

The docking data concerning the interaction between SAC and the TIR domain of TLR4 are shown in Figure 8A,B.

To further strength the reliability of the proposed interaction, the computed model was submitted to 50 ns of all-atom unbiased molecular dynamics simulation. During the simulation, the interactions between SAC and chain A of TIR was well retained (average number of H bonds between SAC and TIR-A: 2.97; Figure 8C), whereas a marked reduction in the interchain number of hydrogen bonds was noted (average number of H bonds between apo-TIR-A and apo-TIR-B: 7.75; average number of H bonds between SAC-bound TIR-A and SAC-bound TIR-B: 4.04; Figure 8D). We thus argued that the binding of SAC led to a destabilization of the TIR-TLR4 dimer, as can be seen by the comparison between Cα-RMSD values of apo-TIR and SAC-bound TIR dimers (Figure 8E,F), and that this effect can be the leading cause of the anti-inflammatory effect of SAC.

Concerning the potential interaction between SAC and TLR2, our data suggest that SAC is expected to disrupt the TLR2/TLR4 heterodimer complex, in virtue of its efficient binding to TLR4. 

## 3. Discussion

One of the clinical features of COVID-19 is the hyperinflammatory activity that is characterized by high expression of IL-6, IL-8 and several other cytokines, chemokines and growth factors [24]. This hyperinflammatory activity is associated with severe forms of COVID-19 and poor prognosis for patients [20]. For instance, Del Valle et al. found that high serum IL-6, IL-8 and TNF-α levels at the time of hospitalization are strong and independent predictors of patient survival [21]. In another study, Burke et al. found that inflammatory phenotyping (revealing upregulation of IL-6 and IL-8 gene expression) predicts clinical outcome in COVID-19 subjects [19]. Therefore, anti-inflammatory compounds and specific clinical protocols are highly needed. Concerning this issue, differential approaches targeting IL-6 and IL-8 cytokines has been proposed in several studies as well as in clinical trials [29,30,31,67,68,69]. Our study demonstrates that the BNT162b2 vaccine induced the NF-kB-dependent IL-8 genes. Studies on the effects of the BNT162b2 vaccine on NF-kB, IL-6, IL-1β, G-CSF, G-CSF and other pro-inflammatory genes are ongoing in our laboratory.

Concerning the effects of the BNT162b2 vaccine on NF-kB and NF-kB-dependent genes, these are expected, considering the effects of purified SARS-CoV-2 S-protein on IB3-1 cells (see Figure 3). In this respect, we should consider that the BNT162b2 vaccine is constituted by a liposomal vaccine vector for carrying the spike mRNA. Since lipid-based nanoparticles are known to stimulate inflammatory responses through endosomal TLR-7 and TLR-8 pathways [70,71,72], we suggest that both lipid formulation and spike mRNA contribute to the activity of BNT162b2 on the expression of pro-inflammatory genes. In any case, in the experiments based on the use of the BNT162b2 vaccine on IB3-1 cells, the effects of anti-inflammatory compounds can be studied on pro-inflammatory genes activated by the BNT162b2 treatment. Interestingly, in most cases, the inducing effects of BNT162b2 treatment on IB3-1 are higher than those of other control stimuli, such as SARS-CoV-2 spike [38,39], Pseudomonas aeruginosa infection [73], TNF-α [74] and LPS [75]. 

The results presented in our study are a proof of principle that the expression of IL-1β, IL-6 and IL-8 genes, coding key proteins of the COVID-19 “cytokine storm” [22], can be inhibited by aged garlic extract (AGE) and the AGE bioactive compound S-allyl-cysteine (SAC). Since the control of the “cytokine storm” is a major issue in the management of COVID-19 patients [76], our study could stimulate research activity that can contribute to the development of protocols useful to control the hyperinflammatory state associated with SARS-CoV-2 infection. This paper is expected to sustain research activity on plant extracts and food supplement containing S-allyl-cysteine and related bioactive molecules in order to support the integration of ‘phyto-preparations’ into conventional/official medicine focusing on COVID-19 treatment. Testing AGE and SAC on SARS-CoV-2-infected cell lines will clarify whether AGE and SAC also exhibit antiviral effects, as we found through using the sulfur compound sulforaphane that they first inhibited the spike-induced expression of NF-kB and NF-kB-regulated genes [38] and then exhibited the inhibition of SARS-CoV-2 infection of bronchial epithelial Calu-3 cells [51]. These pre-clinical investigations are required to propose AGE and SAC in clinical trials. Interest in the possible use of natural products for COVID-19 treatment is demonstrated by ongoing clinical trials, such as the NCT05082727 clinical study based on a garlic-based biomedicine [Safety and Efficacy of Softgel KOVIR (TD0068) in the Combination Regimen With Background Treatment in COVID-19 Patients (KOVIR)] [77]. In the case that the results of our pilot exploratory study will be confirmed and reinforced, then AGE, SAC and other bioactive constituents can be proposed for clinical trials. 

One of the limits of our study is that the activity of AGE and SAC was studied on only three pro-inflammatory genes, IL-1β, IL-6 and IL-8. In this respect, it should be noted that the COVID-19 “cytokine storm” is composed of a large number of pro-inflammatory proteins [19,20,21,22,24]. Therefore, our study is expected to stimulate research activity focusing on the effects of AGE and AGE-related compound on other “cytokine storm” actors, such as IL-2, IL-7, IL-10, G-CSF, GM-CSF, IP10, MCP-1, MIP1A and TNF-α. In addition, only SAC, among the large number of constituents of AGE, was considered. In future experimental efforts, other AGE constituents should be studied, such as allicin, S-allylmercaptocysteine (SAMC) and S-1-propenylcysteine (S1PC) [16,17,18]. 

**Figure 9:**
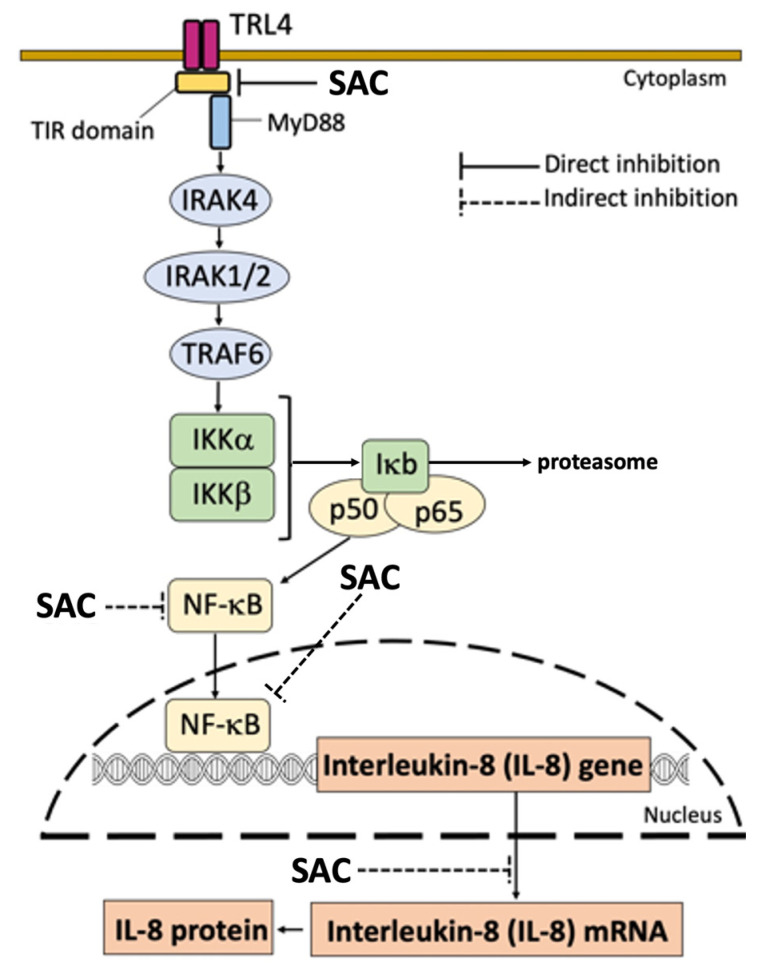
Proposed mechanism of action of SAC. Indirect inhibition of NF-κB might be caused by a direct inhibition of TLR4. The associated NF-κB inhibition is causative of the indirect inhibitory effects of SAC on NF-κB-regulated genes, such as IL-1κ, IL-6, IL-8 and others.

The final limit of our study is that the SAC mechanism of action was not fully analyzed and validated. This should be considered a major issue in the research on this topic in the future since it could help in finding new targets for therapeutic protocols. Among several possibilities (that in any case are not mutually exclusive), SAC can exert its anti-inflammatory activity by NF-κB inhibition. In this context, we have hypothesized that SAC interacts with Toll-like receptors (mainly TLR4). This is shown in Figure 9, which suggest that SAC interacts with and possibly inhibits TLR4 by destabilizing the dimer interactions. Since TLRs are involved in the NF-κB signal transduction [55], SAC might indirectly inhibit NF-κB through a direct inhibition of TLR activity.

In unstimulated cells (i.e., IB3-1 cells cultured in the absence of external stimulation), an inactive trimer is formed in the cytoplasm due to the fact that the NF-κB protein p50/p65 binds the inhibitory protein IκB. In this condition, NF-κB is not translocated to the nucleus. By sharp contrast, when external stimuli act on the corresponding receptors (for example, when TLR4 is activated by SARS-CoV-2 through S-protein/TLR4 interactions), activation of IκK occurs, leading to phosphorylation of IκB. Consequently, phospho-IκB dissociates from the trimer, undergoes proteasome-mediated degradation, and NF-κB is “activated”. In these conditions, the p50/p65 NF-κB proteins translocate to the nucleus and specifically interact with NF-κB binding sites present in the promoters of NF-κB-regulated genes, such as the IL-8 gene (and other genes coding pro-inflammatory proteins) [78,79,80], thus causing transcriptional activation. In addition, upregulated inflammatory factors further activate NF-κB, thereby forming a positive feedback loop. Our hypothesis is that SAC indirectly inhibits the NF-κB pathway (including transcription of the IL-8 gene) through direct inhibition of TRL4. (Figure 9). The interplay of TLR4 and SARS-CoV-2, contributing to the complex mechanisms of inflammation and severity in COVID-19 infections, has been recently reviewed by Asaba et al. [63].

Another member of the Toll-like receptor that should in the future be analyzed for possible interaction(s) with SAC is TLR2. In fact, it is well established that SARS-CoV-2 spike protein induces inflammation and activation of the NF-kB pathway depending on TLR2 [46,81]. In this respect, Kan et al. convincingly reported that the SARS-CoV-2 spike protein induced, as expected, IL-6, TNF-α, and IL-1β in wild-type mice but failed to induce these proteins in TLR2-deficient mice [46]. In addition, when complex geometries will be clarified for other TLR heterodimers (e.g., TLR2/TLR1, TLR2/TLR6), deeper investigations on the potential interaction of SAC with other TLR members will be possible. This might help to better characterize the biological activity of SAC. In this respect, additional experiments which include TLR4-specific ligands, TLR4 inhibitors and TLR2 inhibitors, as well as TLR4 and TLR2 knockout cells will help to validate the model proposed in Figure 9. 

Full understanding of the mechanism of action of SAC will be a very important step to identify novel molecular targets and to develop combined treatments with other anti-inflammatory agents.

## 4. Materials and Methods

### 4.1. Materials

All reagents and chemicals were analytical grade. SARS-Cov-2 spike recombinant glycoprotein (ab49046) was purchased by Abcam (Cambridge, UK). The purity was >90%, as determined by SDS-PAGE. The BNT162b2 vaccine (COMIRNATYTM, Lot. FP8191) was obtained from the Hospital Pharmacy of University of Padova (Roma, Italy).

### 4.2. AGE Extraction and Chemical Characterization

AGE and SAC were provided by Wakunaga Pharmaceutical Co., Ltd. (Hiroshima, Japan) and manufactured as described by Kanamori et al. [11]. Briefly, AGE was prepared by rinsing garlic (*Allium sativum* L.) cloves with purified water, slicing them and then soaking them in a water–ethanol mixture, which was then naturally extracted/aged for more than 10 months at room temperature [28]. The AGE powder used in our experiments was prepared by lyophilization. It contained approximately 28.6% (*w*/*v*, 286 mg/mL) solid material, 0.63% (6.3 mg/mL) arginine and 0.1% SAC (calculated on a dry-weight basis) as a marker compound for standardization [29]. Both SAC and AGE powders were freshly dissolved in complete cell growth medium prior to each experiment. For chemical characterization, AGE and SAC were analyzed by GC-MS as TBDMS derivatives according to Jiménez-Martín et al. [82] and described in detail in the Supplementary Materials. All the reagents have been purchased from Merk (D-6100 Darmstadt, Germany). 

### 4.3. Cell Culture Conditions

The human bronchial epithelial IB3-1 cell line [38] was obtained from LGC Promochem, Europe (Teddington, Middlesex, UK) and cultured in humidified atmosphere as elsewhere described [38]. 

### 4.4. Stimulation of Cells with SARS-CoV-2 Spike Protein

SARS-CoV-2 spike protein (139 KDa, ab49046) was purchased by Abcam (Cambridge, UK). A stock concentration of the protein (7.2 μM in 9% urea, 0.32% Tris-HCl pH 7.2, 50% glycerol) was diluted in 200 µL of LHC-8 medium to achieve the final concentrations used to treat IB3-1 cells [39,40]. Briefly, cells seeded at 50% of confluence were treated with spike protein (5–50 nM) and incubated for 30 min at 4 °C, then for 30 min at 37 °C (this procedure is expected to maximize S-protein interaction with the receptor and the S-protein cellular uptake) [38,39]. After this incubation, LHC-8 medium supplemented with 5% (final concentration) FBS was added to a final 500 μL volume, and the cultures were further incubated at 37 °C and for 24 h.

### 4.5. Treatment of IB3-1 Cells with the BNT162b2 Vaccine

For treatment with the BNT162b2 vaccine, IB3-1 cells were seeded at 50% of confluence and then treated with 1 μg/mL of the vaccine, as elsewhere described [40,41]. After 24 h, cells were additionally treated with AGE or SAC.

### 4.6. RNA Extraction

Cultured cells were trypsinized (0.05% trypsin and 0.02% EDTA; Sigma-Aldrich, Saint Louis, MO, USA) and collected by centrifugation at 1000× *g* for 8 min at 4 °C, washed twice with DPBS 1X (Gibco, Thermo Fischer Scientific, Waltham, MA, USA) and lysed with Tri-Reagent (Sigma-Aldrich), according to the manufacturer’s instructions. The isolated RNA was washed once with cold 75% ethanol, dried and dissolved in nuclease-free pure water before use. Obtained RNA was stored at −80 °C until use [38,83].

### 4.7. Quantitative Analyses of mRNAs

For ILs mRNA analysis, real-time RT-qPCR was performed as described by Gasparello et al. [48]. The experiments were carried out using an assay composed by a primer pair and a fluorescently labeled 5′ nuclease probe purchased from IDT (Integrated DNA Technologies, Coralville, IO, USA; Assays ID: Hs.PT.58.38869678.g for IL-8, Hs.PT.58.40226675 for IL-6 and Hs.PT58.1515156 for IL-1β). Details have been included in Supplementary Materials [38,83].

### 4.8. Analysis of Cytokines, Chemokines and Growth Factors

Proteins released into culture supernatants were measured using Bio-Plex Human Cytokine 27-plex Assay (Bio-Rad, Hercules, CA, USA), as suggested by the manufacturer and described in the Supplementary Materials and in Penolazzi et al. [50].

### 4.9. Effects on Cellular Viability and Apoptosis

Annexin V and dead cell assay was performed using a Muse Cell Analyzer (Merck Millipore) instrument, according to the instructions supplied by the manufacturer. Cells were washed with sterile DPBS 1X, trypsinized, suspended and diluted (1:2) with Muse Annexin V & Dead Cell Reagent (Merck Millipore, St. Louis, MO, USA). After incubation for 15 min at room temperature in the dark, samples were acquired and data were analyzed using Annexin V and Dead Cell Software Module (Merck Millipore) (www.luminexcorp.com/flowkits, accessed on 10 December 2024) [47,48].

### 4.10. Computational Studies

Computational simulations were run on an Ubuntu 20.04 Linux workstation equipped with 32 Core AMD Ryzen 9 with Nvidia Quadro RTX 4000 GPU. The structure of the intracellular domains (TIR) of the TLR4 dimer was derived from the literature [84]. The structure of SAC was prepared with Avogadro software (version 1.95) [85]. A blind docking simulation was performed on the entire TIR dimer surface using AutoDock Vina software [59]. The top-scored complex was submitted to an all-atom unbiased molecular dynamics (MDs) simulation using GROMACS software [86] patched with Plumed v. 2.6.5 [87] under the Charmm36 force field [88], as described in Zurlo et al. [40]. The complex was included in a rectangular box 8 × 10 × 7 nanometers in length, solvated and neutralized using 0.15 M sodium chloride. The full system was submitted to energy minimization and equilibrated under NVT and NPT conditions. Long-range electrostatic interactions were modeled using the particle-mesh Ewald algorithm. LINCS, Nosé-Hoover and Parrinello-Rahman algorithms were used in the simulations for restraints and as thermostat and barostat, respectively. MDs were conducted under the NPT conditions for 50 ns with 2 fs time steps. Root-mean-squared deviation (RMSD) and number of hydrogen bonds were obtained through the “rms” and “hbond” routines implemented in Gromacs.

### 4.11. Statistics

All the data were normally distributed and presented, unless otherwise stated, as mean ± S.D. Statistical differences between groups were compared using one-way ANOVA (analyses of variance between groups) followed by Dunnett’s multiple comparison or paired *t*-test employing Prism (v. 9.02) by GraphPad software. Statistical differences were considered significant when *p* < 0.05 (*) and highly significant when *p* < 0.01 (**) and *p* < 0.001 (***).

## 5. Conclusions

We have developed simple experimental systems and analytical protocols for the screening of molecules interfering with the expression of proteins known to be involved in the COVID-19 “cytokine storm” (Figure 1). The results presented here demonstrate that exposure of epithelial IB3-1 cells to the SARS-CoV-2 spike protein or COVID-19 BNT162b2 vaccine induces increased expression of NF-κB and increased expression of pro-inflammatory genes, particularly of IL-6 and IL-8. Treatment with aged garlic extracts (AGE) and the AGE constituent S-allyl-cysteine (SAC) reverses IL-6 and IL-8 upregulation induced by the SARS-CoV-2 spike protein and BNT162b2 vaccine in IB3-1 cells. Therefore, AGE and AGE constituents should be further evaluated as potential inhibitors of the COVID-19 “cytokine storm”. Further experiments should be programmed to identify other AGE-derived agents able to inhibit changes in gene expression induced by SARS-CoV-2 spike and the COVID-19 BNT162b2 vaccine. Understanding the SAC mechanism of action will help in designing combined therapies with other anti-inflammatory agents. In terms of possible interest to clinical researchers, in case the results of our exploratory pilot study will be confirmed and reinforced on SARS-CoV2-infected cells, then AGE, SAC and other bioactive constituents can be proposed for clinical trials. This might be relevant not only for COVID-19 treatment but also for patients affected by long COVID [89] and post-acute sequelae of SARS-CoV-2 infection (PASC) [90]. 

## Data Availability

All the data will be made available upon request to the corresponding authors.

## Supplementary Materials

The following supporting information can be downloaded at: https://www.mdpi.com/article/10.3390/molecules29245938/s1.
