# Supplementary material for: Aged Garlic Extract (AGE) and Its Constituent S-Allyl-Cysteine (SAC) Inhibit the Expression of Pro-Inflammatory Genes Induced in Bronchial Epithelial IB3-1 Cells by Exposure to the SARS-CoV-2 Spike Protein and the BNT162b2 Vaccine
Source: Molecules. 2024 Dec 16;29(24):5938. doi: 10.3390/molecules29245938 (PMC11677098; doi:10.3390/molecules29245938)
Supplement: Supplementary file 1 [file molecules-29-05938-s001.zip › molecules-3318493-supplementary.pdf]

molecules

Article

Aged Garlic Extract (AGE) and Its Constituent S-Allyl-Cysteine (SAC) Inhibit the Expression of Pro-Inflammatory Genes Induced in Bronchial Epithelial IB3-1 Cells by Exposure to the SARS-CoV-2 Spike Protein and the BNT162b2 Vaccine

Jessica Gasparello [1], Chiara Papi [1], Giovanni Marzaro [2], Alberto Macone [3], Matteo Zurlo [1], Alessia Finotti [2], Enzo Agostinelli [4,5,] * and Roberto Gambari [1,*]

[1] Department of Life Sciences and Biotechnology, Ferrara University; Jessica.Gasparello@gmail.com (J.G.), chiara.papi@unife.it (C.P.), matteo.zurlo@unife,it (M.Z.), alessia.finotti@unife,it (A.F.), gam@unife.it (R.G.)
[2] Department of Diagnostics and Public Health, University of Verona, 37134 Verona, Italy; Giovanni.marzaro@univr.it (G.M.)
[3] Department of Biochemical Sciences 'A. Rossi Fanelli', Sapienza University of Rome, 00185 Rome, Italy; alberto.macone@uniroma1.it (A.M.)
[4] Department of Sensory Organs, Sapienza University of Rome, Policlinico Umberto I, Viale del Policlinico 155, 00161 Rome, Italy; enzo.agostinelli@uniroma1.it (E.A.)
[5] International Polyamines Foundation 'ETS-ONLUS', Via del Forte Tiburtino 98, 00159 Rome, Italy; enzo.agostinelli@uniroma1.it (E.A.)
* Correspondence: gam@unife.it (R.G.), enzo.agostinelli@uniroma1.it (E.A.)

SUPPLEMENTARY MATERIALS

1. Supplementary Materials and Methods

1.1. Chemical characterization of AGE

For chemical characterization, AGE and SAC were analyzed by GC-MS as TBDMS derivatives according to Jiménez-Martín et al. [1]. S-allyl-cysteine (SAC) was dissolved in 0.1 N HCl at a final concentration of 4 mg/mL. In total, 5 μL of these solutions were spiked With 10 μL of internal standard (3,4-dimethoxybenzoic acid, 0.1 mg/mL) and dried under N2. Then, 30 μL of pure MTBSTFA, followed by 30 μL of pyridine, was added. The mixture was heated at 95 °C for 2 hours. The sample was then neutralized with sodium bicarbonate and subjected to GC-MS analysis. The same derivatization protocol was used for AGE power (4 mg/mL 0.1 M HCl). GC-MS analyses were performed with an Agilent 7890B gas chromatograph coupled to a 5977B quadrupole mass selective detector (Agilent Technologies, Palo Alto, CA, USA). Chromatographic separations were carried out with an Agilent HP5ms fused-silica capillary column (30 m × 0.25 mm i.d.) coated with 5%- phenyl-95%-dimethylpolysiloxane (film thickness 0.25 μm) as stationary phase. Injection mode: splitless at a temperature of 280 °C. Column temperature program: 70 °C (1 min) then to 300 °C at a rate of 20 °C/min and held for 10 min. The carrier gas was helium at a constant flow of 1.0 ml/min. The spectra were obtained in the electron impact mode at 70 eV ionization energy; ion source 280 °C; ion source vacuum 10-5 Torr. MS analysis was performed simultaneously in TIC (mass range scan from m/z 50 to 600 at a rate of 0.42 scans s-1) and SIM mode. GC-SIM-MS analysis was performed selecting the following ions: m/z 332 for SAC/S1-PC and m/z 239 for 3,4-dimethoxybenzoic acid (internal standard).

1.2. Stimulation of cells with SARS-CoV-2 Spike protein

SARS-CoV-2 spike protein (139 KDa; stock concentration = 7.2 M in 9% urea, 0.32% Tris-HCl pH 7.2, 50% glycerol) was diluted in 200 μl of LHC-8 medium to achieve the final concentrations used to treat IB3-1 cells. Briefly, cells seeded at 50% of confluence were treated with spike protein (5-50 nM) and incubated for 30 min at 4°C, then for 30 min at 37°C (this procedure is expected to maximize S-protein interaction with the receptor and the S-protein cellular uptake) [2,3]. After this incubation, LHC-8 medium supplemented with 5% (final concentration) FBS was added to a final 500 l volume, and the cultures were further incubated at 37°C for 24h.

1.3. RNA extraction

Cultured cells were trypsinized (0,05% trypsin and 0,02% EDTA; Sigma-Aldrich) and collected by centrifugation at 1,000 x g for 8 min at 4 °C, washed twice with DPBS 1X (Gibco, Thermo Fischer Scientific) and lysed with Tri-Reagent (Sigma-Aldrich), according to the manufacturer’s instructions. The isolated RNA was washed once with cold 75% ethanol, dried and dissolved in nuclease-free pure water before use. Obtained RNA was stored at -80°C until use [2,4].

1.4. Quantitative analyses of mRNAs

For ILs mRNA analysis, 500 ng of total RNA were reverse transcribed to complementary DNA (cDNA) using the Taq-Man Reverse Transcription PCR Kit and random hexamers (Applied Biosystems, Thermo Fischer Scientific) in a final reaction volume of 50 μl. Real-time qPCR experiments were carried out using an assay composed of a primer pair and a fluorescently labeled 5′ nuclease probe purchased from IDT (Integrated DNA Technologies, Coralville, IO, USA; Assays ID: Hs.PT.58.38869678.g for IL-8 and Hs.PT.58.40226675 for IL-6). An amount of 2 μl of cDNA was amplified in the presence of

2x PrimeTime Gene Expression Master Mix for 40 PCR cycles using the CFX96 Touch Real-Time PCR Detection System (Bio-Rad, Hercules, CA, USA). Relative expression was calculated using the comparative cycle threshold method (ΔΔCT method), and the endogenous control human β-actin was used as normalizer. Negative controls (no template cDNA and RT-minus control) were also run in every experimental plate to assess specificity and to rule out contamination. RT-qPCR reactions were performed in duplicate for both target and normalizer genes [2,4].

1.5. Analysis of cytokines, chemokines and growth factors

Proteins released into culture supernatants were measured using Bio-Plex Human Cytokine 27-plex Assay (Bio-Rad), as suggested by the manufacturer. The assay allows the multiplexed quantitative measurement of 27 cytokines/chemokines (including FGF basic, Eotaxin, G-CSF, GM-CSF, IFN-γ, IL-1β, IL-1ra, IL-2, IL-4, IL-5, IL-6, IL-7, IL-8, IL-9, IL-10, IL-12 (p70), IL-13, IL-15, IL-17A, IP-10, MCP-1 (MCAF), MIP-1α, MIP-1β, PDGF-BB, RANTES, TNF-α, VEGF) in a single well. An amount of 50 μl of cytokine standards or samples (diluted supernatants recovered from IB3-1 cells) was incubated with 50 μl of anti-cytokine conjugated beads in a 96-well filter plate for 30 min at room temperature with shaking. The plate was washed by vacuum filtration three times with 100 μl of Bio-Plex Wash Buffer, 25 μl of diluted detection antibody was added to each well, and the plate was incubated for 30 min at room temperature with shaking. After three filter washes, 50 μl of streptavidin-phycoerythrin was added, and the plate was incubated for 10 min at room temperature with shaking. Finally, the plate was washed by vacuum filtration three times, beads were suspended in Bio-Plex Assay Buffer, and the plate was read by a Bio-Rad 96-well plate reader. Collected data were analyzed by the Bio-Plex Manager Software (Bio-Rad) [5].

1.6. Computational studies

All the computational methodologies were carried out on a 32 Core AMD Ryzen 93,905×, 3.5 GHz Linux Workstation (O.S. Ubuntu 20.04) equipped with GPU (Nvidia Quadro RTX 4000, 8 GB). The structure of the intracellular domains (TIR) of the TLR4 dimer was derived from the literature [6]. The structure of SAC was prepared with Avogadro software [7]. Blind docking simulation was performed on the entire TIR dimer surface using AutoDock Vina software [8]. The top-scored complex was submitted to all-atom unbiased molecular dynamics (MDs) simulation using GROMACS software [9] patched with Plumed v. 2.6.5 [10] under the Charmm36 force field [11], as described in Zurlo et al. [12]. The complex was included in a rectangular box 8 x 10 x 7 nanometers in length, solvated and neutralized using 0.15M sodium chloride. The full system was submitted to energy minimization and equilibrated under NVT and NPT conditions. Long-range electrostatic interactions were modeled using the particle-mesh Ewald algorithm. LINCS, Nosé-Hoover and Parrinello-Rahman algorithms were used in the simulations for restraints and as thermostat and barostat, respectively. MDs were conducted under the NPT conditions for 50 ns with 2 fs time steps. Root-mean-squared deviation (RMSD) and number of hydrogen bonds were obtained through the "rms" and "hbond" routines implemented in Gromacs.

2. Supplementary Figure S1

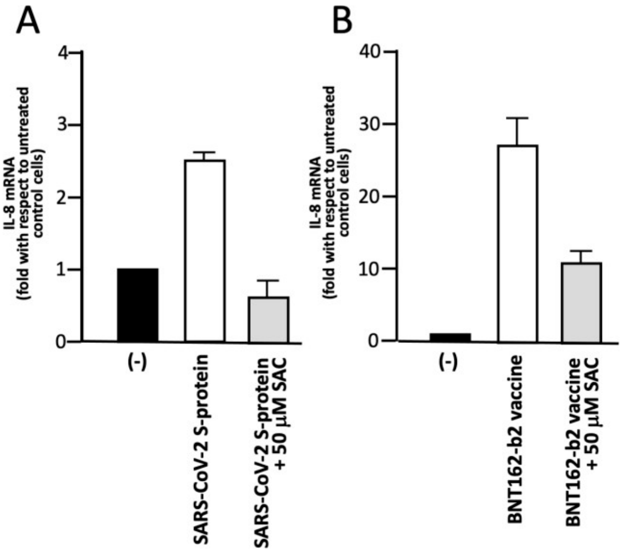

Induction of IL-8 mRNA by bronchial epithelial IB3-1 cells exposed to **5** nM S-protein (A) and 1 µg/ml lBNT162b2 vaccine (B) in the absence and in the presence of 50 µm S-allyl-cysteine (SAC) obtained by Wakunaga Pharmaceuticals Ltd.. Total RNA was isolated from IB3-1 cells after **72** hours treatment, as indicated. Results represent the average fold values of IL6 mRNA in treated bronchial epithelial IB3-1 cells with respect to untreated cells (three independent experiments).

3. Supplementary Figure S2

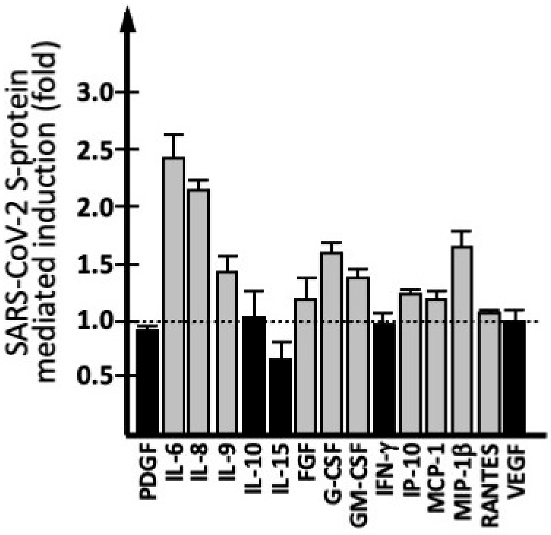

Effects of IB3-1 cell exposure to 5 nM S-protein on released cytokines, chemokines and growth factors. Protein release in supernatants was quantified by Bio-Plex analysis and the ratio of S-protein-treated cells/untreated was calculated. Results represent the average fold values of proteins (pg/ml ± S.D) secreted by S-protein (5 nM) treated bronchial epithelial IB3-1 cells with respect to untreated cells (three independent experiments).

4. Supplementary Table S1.

**Table I. Secreted proteins (characterized by pg/ml values >20 in untreated control IB3-1 cell cultures)**						
	**IL-6**	**IL-8**	**FGF-basic**	**G-CSF**	**RANTES**	**VEGF**
**(-)**	137.84	646.37	32.18	47.69	131.89	116.94
	134.35	633.71	33.21	42.59	132.62	137.24
	139.31	666.36	32.37	41.01	146.36	133.11
	135.15	636.23	32.29	44.36	145.11	119.16
**Average ± SD**	136.66 ± 2.31	645.66 ± 14.84	32.52 ± 0.46	43.94 ± 2.85	138.99 ± 7.81	126.61 ± 10.07
**S-protein**	426.3	1423.93	30.19	74.38	136.62	115.25
	443.1	1531.93	31.02	71.47	131.43	116.22
**Average ± SD**	434.70 ± 11.87	1477.93 ± 76.36	30.61 ± 0.58	72.92 ± 2.06	134.02 ± 3.67	115.73 ± 0.68
**Fold**	3.18	2.29	0.94	1.66	0.96	0.91
***p* value**	< 0.0001	< 0.0001	0.011	0.000235	0.459012	0.223618

Results are from independent experiments.

5. References

1. Jiménez-Martín E, Ruiz J, Pérez-Palacios T, Silva A, Antequera T. Gas chromatography-mass spectrometry method for the determination of free amino acids as their dimethyl-tert-butylsilyl (TBDMS) derivatives in animal source food. *J. Agric. Food Chem.* **2012**, *60*, 2456-63.

2. Gasparello J, D'Aversa E, Papi C, Gambari L, Grigolo B, Borgatti M, Finotti A, Gambari R. Sulforaphane inhibits the expression of interleukin-6 and interleukin-8 induced in bronchial epithelial IB3-1 cells by exposure to the SARS-CoV-2 Spike protein. *Phytomedicine* **2021**, *87*, 153583.

3. Gasparello J, d'Aversa E, Breveglieri G, Borgatti M, Finotti A, Gambari R. In vitro induction of interleukin-8 by SARS-CoV-2 Spike protein is inhibited in bronchial epithelial IB3-1 cells by a miR-93-5p agomiR. *Int. Immunopharmacol.* **2021**, *101*:108201.

4. Gasparello J, Lomazzi M, Papi C, D'Aversa E, Sansone F, Casnati A, Donofrio G, Gambari R, Finotti A. Efficient Delivery of MicroRNA and AntimiRNA Molecules Using an Argininocalix[4]arene Macrocycle. *Mol. Ther. Nucleic Acids.* **2019**, *18*, 748-763

5. Penolazzi L, Lambertini E, Tavanti E, Torreggiani E, Vesce F, Gambari R, Piva R. Evaluation of chemokine and cytokine profiles in osteoblast progenitors from umbilical cord blood stem cells by BIO-PLEX technology. *Cell Biol. Int.* **2008**, *32*, 320-25.

6. Patra MC, Kwon HK, Batool M, Choi S. Computational Insight Into the Structural Organization of Full-Length Toll-Like Receptor 4 Dimer in a Model Phospholipid Bilayer. *Front. Immunol.* **2018**, *9*, 489.

7. Hanwell MD, Curtis DE, Lonie DC, Vandermeersch T, Zurek E, Hutchison GR. Avogadro: an advanced semantic chemical editor, visualization, and analysis platform. *J. Cheminform.* **2012**, *4*, 17.

8. Eberhardt J, Santos-Martins D, Tillack AF, Forli S. AutoDock Vina 1.2.0: New Docking Methods, Expanded Force Field, and Python Bindings. *J. Chem. Inf .Model.* **2021,** *61*, 3891-3898.

9. Abraham MJ, Murtola T, Schulz R, Páll S, Smith JC, Hess B, Lindahl E. GROMACS: High performance molecular simulations through multi-level parallelism from laptops to supercomputers. *SoftwareX* **2015**, *1–2*, 19-25.

10. Bonomi M, Branduardi D, Bussi G, Camilloni C, Provasi D, Raiteri P, Donadio D, Marinelli F, Pietrucci F, Broglia R, Parrinello M. PLUMED: A portable plugin for free-energy calculations with molecular dynamics. *Computer Physics Comm.* **2009**, *180*, 1961-1972, 2009.

11. Huang J, MacKerell AD. CHARMM36 all-atom additive protein force field: Validation based on comparison to NMR data. *J. Comput. Chem.* **2013**, *34*, 2135-2145.

12. Zurlo M, Gasparello J, Verona M, Papi C, Cosenza LC, Finotti A, Marzaro G, Gambari R. The anti-SARS-CoV-2 BNT162b2 vaccine suppresses mithramycin-induced erythroid differentiation and expression of embryo-fetal globin genes in human erythroleukemia K562 cells. *Exp. Cell. Res.* **2023**, *433*, 113853.
